# Identification of sentinel lymph node macrometastasis in breast cancer by deep learning based on clinicopathological characteristics

**DOI:** 10.1038/s41598-024-78040-y

**Published:** 2024-11-06

**Authors:** Daqu Zhang, Miriam Svensson, Patrik Edén, Looket Dihge

**Affiliations:** 1 Division of Computational Science for Health and Environment, Center for Environmental and Climate Science, Lund University, Lund, Sweden; 2 Department of Clinical Sciences Lund, Division of Surgery, Lund University, Lund, Sweden; 3 Department of Plastic and Reconstructive Surgery, Skåne University Hospital, Malmö, Sweden

**Keywords:** Breast cancer, Lymphatic metastasis, Sentinel lymph node, Deep learning, Clinical decision support, Cancer, Computational biology and bioinformatics, Health care, Medical research, Oncology, Breast cancer

## Abstract

The axillary lymph node status remains an important prognostic factor in breast cancer, and nodal staging using sentinel lymph node biopsy (SLNB) is routine. Randomized clinical trials provide evidence supporting de-escalation of axillary surgery and omission of SLNB in patients at low risk. However, identifying sentinel lymph node macrometastases (macro-SLNMs) is crucial for planning treatment tailored to the individual patient. This study is the first to explore the capacity of deep learning (DL) models to identify macro-SLNMs based on preoperative clinicopathological characteristics. We trained and validated five multivariable models using a population-based cohort of 18,185 patients. DL models outperform logistic regression, with Transformer showing the strongest results, under the constraint that the sensitivity is no less than 90%, reflecting the sensitivity of SLNB. This highlights the feasibility of noninvasive macro-SLNM prediction using DL. Feature importance analysis revealed that patients with similar characteristics exhibited different nodal status predictions, indicating the need for additional predictors for further improvement.

## Introduction

Breast cancer is the most common cancer worldwide and one of the leading causes of cancer-related death in women^1^. Along with tumor size and histological grade, axillary lymph node status is one of the strongest prognostic factors for breast cancer^2–4^. Sentinel lymph nodes (SLNs) are the first axillary lymph nodes to receive drainage from a breast tumor; therefore, they are most likely to exhibit metastatic deposits. SLN biopsy (SLNB) remains the gold standard approach for staging the axillary nodal status in patients with clinically node-negative (cN0) breast cancer and also in cN + patients undergoing neoadjuvant chemotherapy with complete pathological response at axillary level^5–7^. In particular, the presence of SLN macrometastasis (macro-SLNM, a metastasis > 2 mm) is clinically significant and affects decision-making in systemic and locoregional therapy^8,9^. In patients with macro-SLNM undergoing mastectomy and immediate breast reconstruction (IBR), post-mastectomy radiotherapy (PMRT) is associated with a high risk of postoperative complications, including implant failure^10^.

While SLNB reduces postoperative arm morbidity and maintains oncological safety comparable to axillary lymph node dissection (ALND)^11,12^, a risk of postoperative complications still remains. When assessing the outcomes of patients with cN0 breast cancer randomized to SLNB or no axillary intervention, SLNB was associated with significantly increased arm and breast morbidity^13^. Considering the low incidence of clinically relevant macro-SLNMs in contemporary breast cancer populations^14–16^, the utility of routine SLNB in all patients with primary invasive breast cancer has been questioned, and there is a current trend toward de-escalation of axillary surgery^17^. Axillary ultrasonography (AUS) enables noninvasive axillary staging and is currently included in the diagnostic work-up for primary breast cancer. When utilizing radiomics and advanced adjunctive modalities, AUS has demonstrated good performance^18,19^. However, AUS is highly operator-dependent, resulting in a wide range of reported accuracy^20,21^. To date, no reliable non-invasive method has been clinically implemented to replace SLNB for staging the axillary nodal status in patients with cN0 breast cancer.

Multiple studies have attempted to noninvasively predict the axillary nodal status in patients with breast cancer using prediction models based on clinicopathological characteristics^22–26^. Although the results are promising, these studies predominantly rely on linear models such as logistic regression (LR), with few studies exploring decision trees and multilayer perceptron (MLP). These traditional machine learning (ML) techniques have limited ability for feature extraction, leaving potential complex feature interactions unexplored. Conversely, deep learning (DL) algorithms harness artificial neural networks with sophisticated architectures and have the advantages of nonlinear modeling and advanced feature engineering. This makes DL invaluable for risk estimation in clinical practice, as it has the potential to capture complex data interactions that other algorithms may miss. Previous studies have shown the immense potential of DL in predicting lymph node metastasis using radiomics data^18,27–29^. However, owing to the restricted accessibility of these data, the usefulness of these prediction tools is limited. By contrast, routinely collected health data often include clinicopathological characteristics, and this wealth of tabular data presents an extraordinary opportunity for DL models. Many encouraging attempts have been made to adapt DL for tabular data^30–32^. In particular, Transformer^33^, which is based on a self-attention mechanism, is effective in capturing global correlations among predictors. A recent benchmarking study on diverse tabular tasks revealed that ResNet^34^ is an effective baseline and that Transformer outperforms other DL solutions on most tasks^35^, although Transformer’s superiority over gradient-boosted decision trees on tabular data is still under debate^36^. Nevertheless, there has been no reported research investigating DL for predicting nodal status in breast cancer based solely on clinicopathological characteristics.

In this study, we aimed to fill the following knowledge gap: what is the full potential of DL for predicting macro-SLNM in cN0 primary invasive breast cancer using solely clinicopathological variables that are easily accessible in a preoperative setting? To this end, we used a large contemporary population-based dataset of 18,185 patients from the Swedish National Quality Registry for Breast Cancer (NKBC)^37^. For the models, we implemented ResNet and Transformer, as well as LR, MLP, and CatBoost^38^ as benchmarks. To take full advantage of these models, we tested several powerful DL strategies on our data, including feature tokenizers^33^ for efficient feature embedding; weighted binary cross-entropy loss, focal loss^39^, and triplet loss^40^ to address the imbalanced distribution of macro-SLNM; and Bayesian optimization of the hyperparameter search. Last, to better visualize and interpret the clinical importance of the included predictors, we utilized Shapley Additive exPlanations (SHAP)^41^ to estimate feature importance.

## Results

### Development and test cohorts show trivial differences in clinical characteristics

A total of 23,264 patients diagnosed with breast cancer between 2014 and 2017 who underwent surgical treatment were identified within the NKBC (Supplementary Fig. S1). Of these, 18,185 were included in the study cohort, with an overall macro-SLNM prevalence of 13%, and the mean number of sentinel nodes harvested was 1.90, with a median of 2.00. In the overall study cohort, 2,185 patients underwent completion ALND. The mean age at diagnosis was 63 years, and most patients had Luminal A-like (LumA) tumors, grade II carcinoma of no special type (NST) with a median tumor size of 16.3 mm. Patients diagnosed between 2014 and 2016 (*n* = 13,656) were used for model development (training and internal validation), and those diagnosed in 2017 were assigned to the test set (*n* = 4529). Thirteen clinical features previously recognized as predictive of the axillary nodal status^42,43^ were used to train the prediction models. A comparison of these features and the outcome (macro-SLNM) between the training and test sets is presented in Table 1. The observed significance could be influenced by the large sample size, which tends to result in high confidence (small* P* values) while not providing direct information about the magnitude of the detected differences. To address this issue, the effect size (*V* or *d*) was also analyzed. In the test set, tumors were more often detected by mammography screening (61 *vs* 58%; *P* = 0.003, *V* = 0.022), there was a slightly lower frequency of invasive lobular carcinoma (ILC) (12 *vs* 13%; *P* < 0.001, *V* = 0.018), and the progesterone receptor (PgR) expression was lower (58.0 *vs* 59.9%; *P* = 0.007, *d* = -0.047). However, there was a higher frequency of the LumA molecular subtype in the test set (59% *vs* 55%; *P* < 0.001, *V* = 0.020). The test set also exhibited a slightly lower prevalence of macro-SLNM (12 *vs* 14%; *P* = 0.030, *V* = 0.016). It is worth noting that none of the cohort differences exhibited non-trivial effect sizes (defined as |*V*|≥ 0.30, ≥ 0.21, and ≥ 0.17 for 1, 2, and 3 degrees of freedom, respectively^44^), indicating that the observed variations between the development and test sets were small.

**Table 1:** Development and test sets show trivial differences in clinical characteristics.

Characteristics	All	Development set	Test set	*P* value	Effect size
	(*n* = 18,185)	(*n* = 13,656)	(*n* = 4,529)		
Age, y, mean (SD)	62.8 (± 12.0)	62.7 (± 12.0)	63.2 (± 11.9)	0.020	0.040
Menstrual status, No. (%)				0.574	0.004
Premenopausal	3280 (19)	2468 (19)	812 (19)		
Postmenopausal	13,857 (81)	10,361 (81)	3496 (81)		
Mode of detection, No. (%)				0.003	0.022
Symptomatic presentation	7502 (41)	5717 (42)	1785 (39)		
Mammographic screening	10,643 (59)	7904 (58)	2739 (61)		
No. Invasive foci, mean (SD)	1.3 (± 0.7)	1.3 (± 0.7)	1.3 (± 0.7)	0.439	0.013
T-stage, No. (%)				0.038	0.015
T1	13,793 (76)	10,306 (75)	3487 (77)		
T2	4392 (24)	3350 (25)	1042 (23)		
Tumor size, mm, mean (SD)	16.3 (± 8.8)	16.3 (± 8.9)	16.1 (± 8.6)	0.066	-0.031
Histological type, No. (%)				< 0.001	0.018
NST	13,979 (77)	10,511 (77)	3468 (77)		
ILC	2320 (13)	1779 (13)	541 (12)		
Others	1315 (7)	977 (7)	338 (7)		
Histological grade, No. (%)				0.081	0.012
I	4068 (23)	3081 (23)	987 (22)		
II	9447 (53)	7118 (53)	2329 (52)		
III	4467 (25)	3298 (24)	1169 (26)		
ER, %, mean (SD)	86.2 (± 29.7)	86.3 (± 29.4)	85,9 (± 30,4)	0.549	-0.011
PgR, %, mean (SD)	59.4 (± 39.4)	59.9 (± 39.2)	58.0 (± 39.9)	0.007	-0.047
Ki67, %, mean (SD)	25.8 (± 19.8)	25.8 (± 19.9)	25.9 (± 19.3)	0.789	0.005
HER2 status, No. (%)				0.631	0.004
Negative	15,917 (89)	11,939 (89)	3978 (89)		
Positive	2009 (11)	1497 (11)	512 (11)		
St Gallen surrogate molecular subtype, No. (%)				< 0.001	0.020
LumA	9589 (56)	7010 (55)	2579 (59)		
LumB	4380 (26)	3362 (26)	1018 (23)		
HER2 +	2009 (12)	1497 (12)	512 (12)		
TNBC	1131 (7)	842 (7)	289 (7)		
# Harvested SLNs, mean (SD)	1.9 (± 1.1)	1.9 (± 1.1)	2.0 (± 1.2)	< 0.001	0.14
Macro-SLNM, No. (%)				0.030	0.016
Negative	15,776 (87)	11,804 (86)	3972 (88)		
Positive	2409 (13)	1852 (14)	557 (12)		

*P* values and effect sizes were calculated for the development *vs* test sets. The significance level was set at *P* = 0.05, and a non-trivial effect size was defined as |*V*|≥ 0.30, ≥ 0.21, and ≥ 0.17 for 1, 2, and 3 degrees of freedom, respectively.

*NST* No special type; *ILC* Invasive lobular carcinoma; *ER* Estrogen receptor; *PgR* Progesterone receptor; *HER*2 Human epidermal growth factor receptor 2; *LumA* luminal A-like; *LumB* Luminal B-like; *HER*2 +  HER2-positive; *TNBC* Triple-negative breast cancer; *SLN* Sentinel lymph node; *macro*-*SLNM* Sentinel lymph node macrometastasis.

### Tumor size and number of invasive foci are significant clinical predictors for macro-SLNM

A comparison of patient and tumor characteristics between patients with and without macro-SLNM in the overall study cohort is presented in Table 2. Patients with macro-SLNM were younger, more frequently premenopausal, and had a higher prevalence of symptomatically presented breast tumors. Furthermore, they exhibited more invasive foci, larger tumor size, higher histological grade, higher expression of Ki67, and a higher rate of the Luminal B-like (LumB) molecular subtype. Although a wide range of variables showed significant differences, only a limited number exhibited nontrivial effect sizes. Significant differences with non-trivial effect sizes were observed for tumor size (21.7 mm *vs* 15.4 mm;* P* < 0.001; *d* = 0.707). Additionally, marginal non-trivial effects were observed for number of invasive foci (1.5 *vs* 1.2; *P* < 0.001; *d* = 0.333), Ki67 expression (28.0 *vs* 25.5%; *P* < 0.001; *d* = 0.129) and T stage > T1 (45% *vs* 21%;* P* < 0.001; *V* = 0.191).

**Table 2:** Tumor size and number of invasive foci are significant clinical predictors of sentinel lymph node macrometastasis (macro-SLNM).

Characteristics	All	Macro-SLNM		*P* value	Effect size
	(*n* = 18,185)	Negative (*n* = 15,776)	Positive (*n* = 2,409)		
Age, y, mean (SD)	62.8 (± 12.0)	62.9 (± 11.8)	62.3 (± 13.0)	0.035	-0.049
Menstrual status, No. (%)				< 0.001	0.038
Premenopausal	3280 (19)	2757 (19)	523 (23)		
Postmenopausal	13,857 (81)	12,104 (81)	1753 (77)		
Mode of detection, No. (%)				< 0.001	0.084
Symptomatic presentation	7502 (41)	6254 (40)	1,248 (52)		
Mammographic screening	10,643 (59)	9485 (60)	1,158 (48)		
No. Invasive foci, mean (SD)	1.3 (± 0.7)	1.2 (± 0.6)	1.5 (± 0.9)	< 0.001	0.333
T-stage, No. (%)				< 0.001	0.191
T1	13,793 (76)	12,469 (79)	1324 (55)		
T2	4392 (24)	3307 (21)	1085 (45)		
Tumor size, mm, mean (SD)	16.3 (± 8.8)	15.4 (± 8.3)	21.7 (± 9.9)	< 0.001	0.707
Histological type, No. (%)				< 0.001	0.036
NST	13,979 (77)	12,108 (77)	1871 (78)		
ILC	2320 (13)	1962 (12)	358 (15)		
Others	1315 (7)	1232 (8)	83 (3)		
Histological grade, No. (%)				< 0.001	0.068
I	4068 (23)	3760 (24)	308 (13)		
II	9447 (53)	8109 (52)	1338 (56)		
III	4467 (25)	3726 (24)	741 (31)		
ER, %, mean (SD)	86.2 (± 29.7)	86.2 (± 29.7)	85.8 (± 29.5)	0.481	-0.016
PgR, %, mean (SD)	59.4 (± 39.4)	59.5 (± 39.5)	59.1 (± 38.7)	0.674	-0.009
Ki67, %, mean (SD)	25.8 (± 19.8)	25.5 (± 19.8)	28.0 (± 19.3)	< 0.001	0.129
HER2 status, No. (%)				0.801	0.002
Negative	15,917 (89)	13,810 (89)	2107 (89)		
Positive	2009 (11)	1739 (11)	270 (11)		
St Gallen surrogate molecular subtype, No. (%)				< 0.001	0.049
LumA	9589 (56)	8518 (57)	1071 (47)		
LumB	4380 (26)	3588 (24)	792 (35)		
HER2 +	2009 (12)	1739 (12)	270 (12)		
TNBC	1131 (7)	989 (7)	142 (6)		

*P* values and effect sizes were calculated for negative *vs* positive macro-SLNM. The significance level was set at *P* = 0.05, and a non-trivial effect size was defined as |*V*|≥ 0.30, ≥ 0.21, and ≥ 0.17 for 1, 2, and 3 degrees of freedom, respectively.

*NST* No special type; *ILC* Invasive lobular carcinoma; *ER* Estrogen receptor; *PgR* Progesterone receptor; *HER*2 Human epidermal growth factor receptor 2; *LumA* luminal A-like; *LumB* Luminal B-like; *HER*2 +  HER2-positive; *TNBC* Triple-negative breast cancer.

### LR and MLP exhibit weak advantages over Transformer and outperform the remaining models on overall performance

**Fig. 1:**
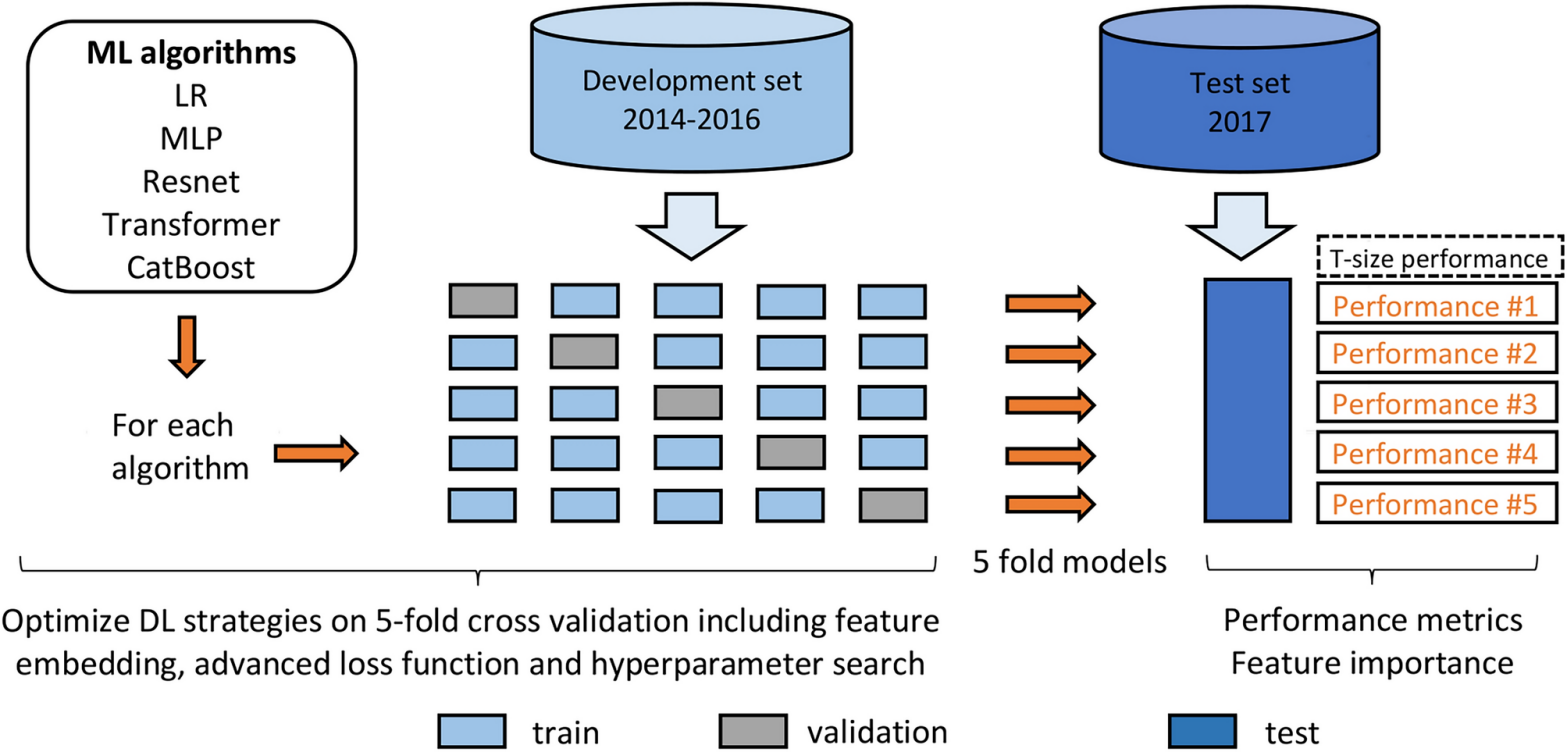
Deep learning workflow. The overall study cohort was divided into a development set (patients diagnosed between 2014 and 2016) and a test set (patients diagnosed in 2017). Five multivariable machine learning (ML) algorithms (logistic regression [LR], multilayer perceptron [MLP], ResNet, Transformer, and CatBoost) were trained on the development set to predict sentinel lymph node macrometastasis (macro-SLNM, a metastasis > 2 mm). Several powerful deep learning (DL) strategies were employed to take full advantage of the prediction models, including feature tokenizers for efficient feature embedding; weighted binary cross-entropy loss, focal loss, and triplet loss to address the imbalanced distribution of macro-SLNMs; and Bayesian optimization of the hyperparameter search. Internal validation was performed using fivefold cross validation. The trained fivefold models of each multivariable algorithm and the univariable model using only tumor size (T-size) were evaluated on the test set to estimate predictive performance. Performance metrics, including the area under the receiver operating characteristic (ROC) curve (AUC) and the precision recall (PR) AUC, were calculated. In addition, when the sensitivity was set to at least 90%, the specificity, negative predictive value, and positive predictive value were calculated based on the average performance across all five folds. Finally, Shapley Additive exPlanations was applied to evaluate the feature importance for each of the five multivariable algorithms and for individual patients.

**Fig. 2:**
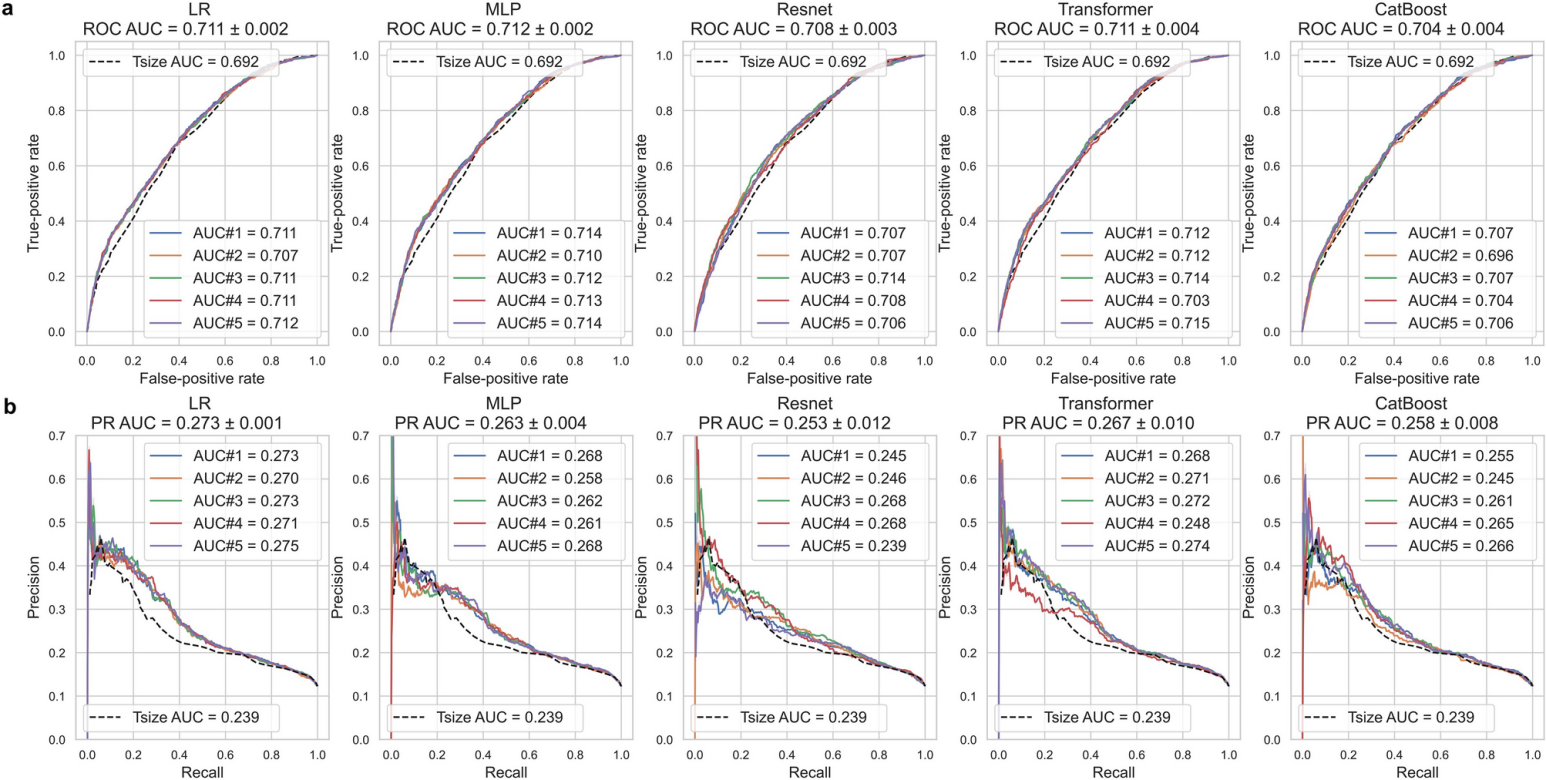
Logistic regression (LR) and multilayer perceptron (MLP) exhibit weak advantages over Transformer and outperform the remaining models on overall performance. (**A**) Receiver operating characteristic (ROC) and (**B**) precision recall (PR) curves for fivefold models of all multivariable algorithms on the test set. The ROC/PR curves of the univariable model based on only tumor size (T-size) serve as a shared benchmark (dashed line in black). Presented at the top is the mean area under the curve (AUC) and standard deviation across all 5 folds.

To investigate the added value of DL models in predicting macro-SLNM using only preoperatively accessible variables, we first benchmarked a univariable model (called T-size), using only tumor size, as it was previously verified as one of the most important predictors of axillary nodal status.^45^ We then compared the overall performance of the five multivariable models trained on the 13 clinical features (Supplementary Table S1). The detailed DL workflow is illustrated in Fig. 1. The models were evaluated using the area under the receiver operating characteristic (ROC) curve (AUC). We also used precision-recall (PR) AUC, which is recommended for data with imbalanced classes. Figure 2(A) and (B) demonstrate the predictive ability of the five multivariable ML models and the univariable T-size model on the test set. With regard to the ROC AUC, Transformer, LR, and MLP exhibited similar performances and did not show substantial differences (range 0.711–0.712). These models slightly outperformed ResNet and CatBoost (range 0.704–0.708). For the PR AUC, LR had the highest performance (0.273 ± 0.001), albeit with a marginal advantage < 0.010 compared to MLP and Transformer. Surprisingly, the improvement observed across all the developed ML models was minimal compared to the univariable T-size model, with only slight increases of 2.0% in the ROC AUC and 3.4% in the PR AUC.

### DL models outperform LR under the constraint that the sensitivity is no less than 90%

The procedural accuracy of SLNB is assessed by calculating the false-negative rate (FNR), with a generally accepted value of 10%^46^. Therefore, we also optimized the decision thresholds for each developed model by maximizing the specificity while ensuring a sensitivity of at least 90%. Table 3 summarizes the corresponding performance metrics for the specified thresholds. The mean and standard deviations were calculated across the fivefold prediction models tested on the test set. Transformer had the highest specificity (34.6 ± 0.6%), precision (16.2 ± 0.1%), negative predictive value (NPV) (96.2 ± 0.1%), and accuracy (41.5 ± 0.5%). MLP also showed competitive performance. Interestingly, although LR had a better overall performance in terms of the PR AUC, all other ML models outperformed LR at 90% sensitivity and had higher specificity, precision, NPV, and accuracy. For further details, the distribution of individual predictions by Transformer are shown in Supplementary Fig. S2.

**Table 3:** Deep learning models outperform logistic regression (LR) under the constraint that sensitivity is no less than 90%.

	T-size	Multivariable models				
		LR	MLP	Resnet	Transformer	CatBoost
ROC AUC	0.692	0.711 (± 0.002)	**0.712 (± 0.002)**	0.708 (± 0.003)	0.711 (± 0.004)	0.704 (± 0.004)
PR AUC	0.239	**0.273 (± 0.001)**	0.263 (± 0.004)	0.253 (± 0.012)	0.267 (± 0.010)	0.258 (± 0.008)
Sensitivity (recall TPR), %	90.1	90.1	90.1	90.1	90.1	90.1
Specificity (TNR), %	31.8	32.6 (± 0.5)	34.2 (± 0.9)	33.0 (± 0.7)	**34.6 (± 0.6)**	32.8 (± 1.3)
PPV (precision), %	15.6	15.8 (± 0.1)	16.1 (± 0.2)	15.9 (± 0.1)	**16.2 (± 0.1)**	15.8 (± 0.2)
NPV, %	95.8	95.9 (± 0.1)	96.1 (± 0.1)	96.0 (± 0.1)	**96.2 (± 0.1)**	95.9 (± 0.2)
Accuracy, %	39.0	39.7 (± 0.4)	41.0 (± 0.8)	40.0 (± 0.6)	**41.5 (± 0.5)**	39.9 (± 1.1)

The performance metrics of the multivariable models were evaluated using the test set by calculating the mean and standard deviation across the fivefold models. The specificity, positive predictive value (PPV), negative predictive value (NPV), and accuracy at a sensitivity threshold of no less than 90% were presented. The best results for each metric are indicated in bold.

*T-size* Tumor size; *MLP* Multilayer perceptron; *ROC* Receiver operating characteristic; *AUC* Area under the curve; *PR* Precision recall; *TPR* True positive rate; *TNR* True negative rate.

### Tumor size shows a significant lead over the other predictors

**Fig. 3:**
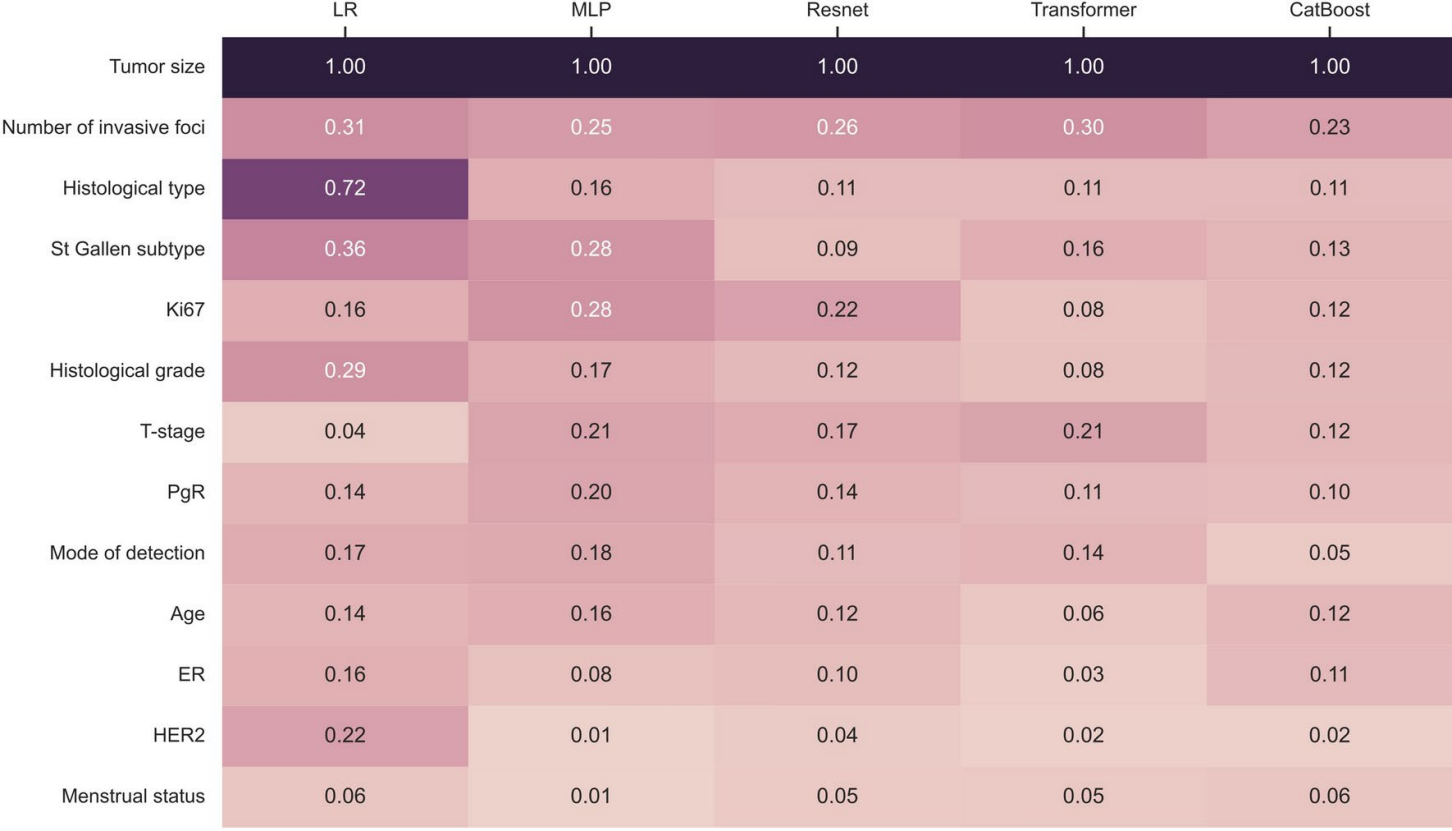
Tumor size shows a significant lead over the other predictors. A heat map of the feature importance assessed by Shapley Additive exPlanations (SHAP) is presented for each of the five multivariable models. The predictors are ranked by average importance across all models, with decreasing values from top to bottom. LR, logistic regression; MLP, multilayer perceptron; PgR, progesterone receptor; ER, estrogen receptor; HER2, human epidermal growth factor receptor 2.

Finally, we employed the SHAP explainer to estimate the feature importance of the multivariable models^41^. In Fig. 3, the predictors are ranked by importance across all models, with decreasing average importance from top to bottom. Tumor size was the single most important factor in all predictive models, and in three of the models, the number of invasive foci was the second most important factor. Notably, tumor size exhibited a significant lead over the second-ranked variable in all predictive models (*P* < 0.001), highlighting its prominent role in predicting macro-SLNM, which is in line with the previous findings^45^. The reliability of the SHAP explainer was verified to be highly consistent with the LR model coefficients (Supplementary Fig. S3).

Among the models, LR demonstrated a distinctive distribution of feature importance. More specifically, LR attributed a substantial importance value (0.72) to the histological type predictor, whereas other models displayed only limited importance for this feature. In addition, LR attributed considerable importance to the human epidermal growth factor 2 (HER2) predictor, ranking it much higher than the other algorithms. Furthermore, when examining the correlation between the values of feature importance assigned by each model and the effect sizes, LR exhibited a large discrepancy, with a correlation coefficient of 0.670, whereas Transformer exhibited the highest consistency, with a correlation of 0.965, closely followed by ResNet and CatBoost.

### Individual interpretations indicate data limitation

**Fig. 4:**
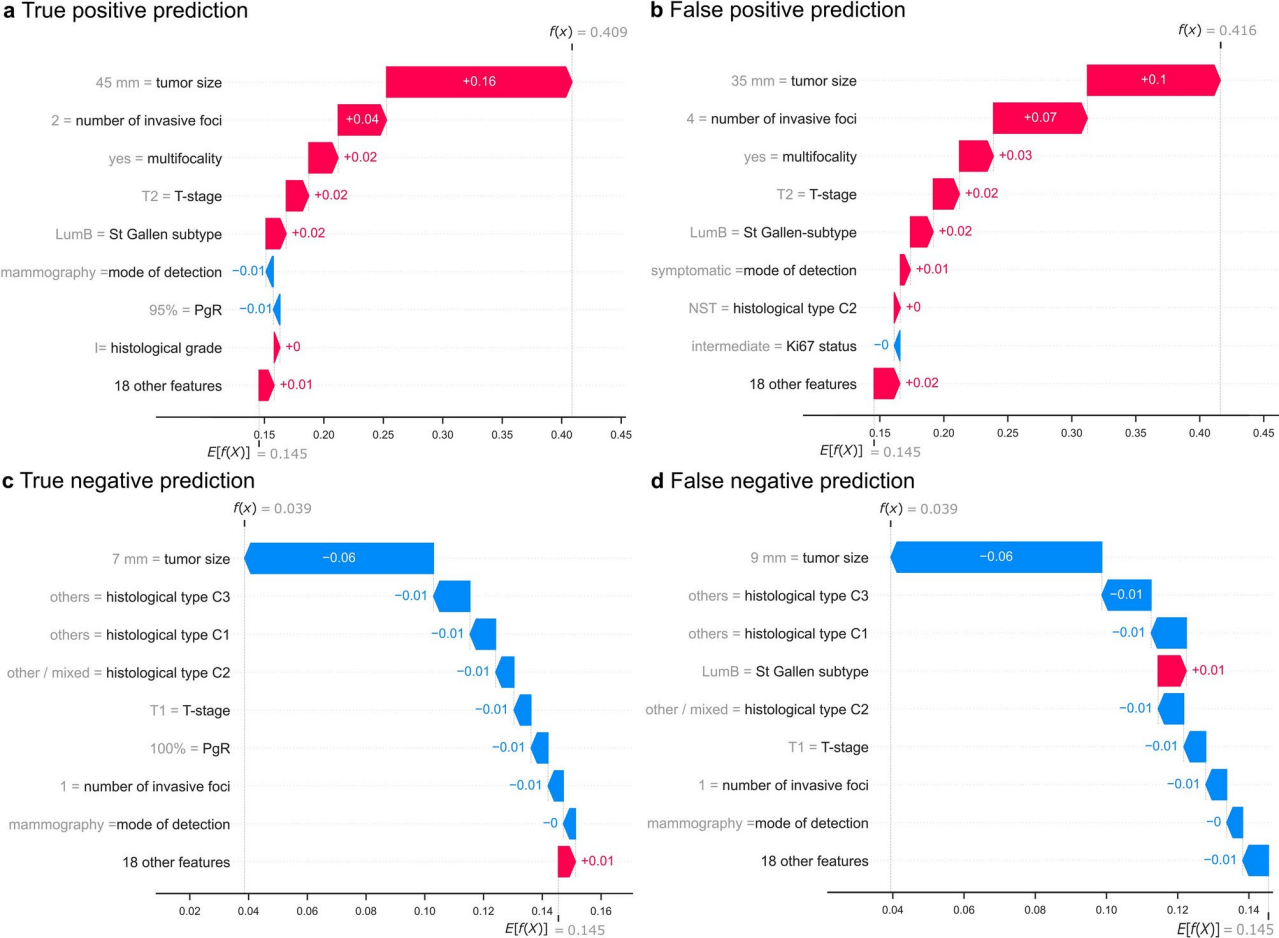
Individual interpretations indicate data limitations. Shown here are the individual Shapley Additive exPlanations (SHAP) of four different patients for the prediction of sentinel lymph node macrometastasis (macro-SLNM) based on the Transformer model. (**A**) True-positive, (**B**) false-positive, (**C**) true-negative, and (**D**) false-negative predictions were randomly selected from the test set. Here, f(x) is the predicted probability of macro-SLNM for the selected patient based on the transformer model, and E[f(x)] is the expectation (mean value) of predictions across the entire test set (0.145), as well as the threshold for positive and negative predictions. For each individual, the prediction starts from E[f(x)], and each predictor contributes positively (red) or negatively (blue) to the final prediction f(x). The predictors are sorted according to the absolute feature importance (contribution) of each predictor. The 26 redundant predictors are shown directly because there may be both negative and positive values within one redundant group (see Supplementary Table S1). LumA, luminal A-like; PgR, progesterone receptor; ILC, invasive lobular carcinoma; NST, no special type; C1, classification 1 [NST, ILC, others]; C2, classification 2 [NST, ILC, other or mixed]; C3, classification 3 [NST or ILC, others].

Based on Transformer’s prediction, we randomly selected true-positive, false-positive, true-negative, and false-negative predictions from the test set (Fig. 4(A–D)) and examined their individual SHAP explanations. For two patients with model-predicted macro-SLNM, both the true-positive prediction (Fig. 4(A)) and the false-positive prediction (Fig. 4(B)) had a large tumor size (45 and 35 mm, respectively) and multifocality (4 and 2 invasive foci, respectively). These two key predictors significantly contributed to positive predictions in these two patients. In contrast, for two patients with model-predicted absence of macro-SLNM, the true-negative prediction (Fig. 4(C)) and the false-negative prediction (Fig. 4(D)) both had a small tumor size (7 and 9 mm, respectively) and unifocality along with a histological type other than NST or ILC. The other models yielded similar results. In summary, some patients with similar features obtained close model predictions although they had different SLNB outcomes, indicating the intricate nature of nodal status prediction and the data limitations of only routine clinicopathological predictors.

### Advanced losses and hyperparameter optimization do not improve the predictive performance

Various strategies, including feature tokenizers, advanced losses, and hyperparameter searches, were explored to enhance the performance of the DL models. Interestingly, the results indicated that, except for the tokenizer, which was found to be essential for Transformer, none of the explored strategies significantly improved the predictive ability in the internal validation (details are shown in Supplementary Figs. S4, S5, S6). Based on these observations, the multivariable models presented above were trained using default hyperparameters (see Supplementary Section B) and optimized for binary cross-entropy loss.

## Discussion

This study extensively explored the capacity of DL models to predict macro-SLNM using 13 clinicopathological features in a contemporary cohort of > 18,000 women with cN0 T1-T2 breast cancer. Compared to traditional ML models, DL did not show significant advantages in terms of overall performance. However, when the tolerance of the FNR was set to 10%, which is the generally accepted FNR of SLNB^46^, Transformer showed superiority, with a specificity of 34.6% (± 0.6%) and an NPV of 96.2% (± 0.1%). The results highlight the feasibility of non-invasive prediction of clinically significant macro-SLNM using DL models but underline that individual-level interpretation has irreducible data uncertainties, which suggests the need for inclusion of additional variables in prediction models to improve their accuracy in further studies.

Artificial intelligence, particularly DL, has recently gained popularity in risk stratification owing to its outstanding performance^47^. Cutting-edge DL techniques have revolutionized the way mammography^48–51^, histopathological slides^52–54^, and gene expression data^55^ are analyzed and interpreted, leading to transformative outcomes in breast cancer. Wang et al.^50^ and Yala et al.^51^ proved that DL models trained on mammography were superior to standard methods for risk discrimination in breast cancer. For the analysis of histopathological slides, DL has demonstrated significant improvement in identifying metastasis in lymph node biopsies^52,53^, as well as in classifying different types of breast cancer.^54^ Moreover, pioneering research has suggested the potential of DL for detecting breast cancer from gene expression data and identifying high-risk genes^55^. In terms of predicting lymph node metastasis, DL models exploiting radiomics, such as magnetic resonance imaging^28^, shear wave elastography,^18^ contrast-enhanced ultrasonography^56^, and positron emission tomography^29^, have demonstrated excellent performance, with ROC AUCs between 0.82 and 0.94. However, these imaging features are not always available in routine breast cancer work-ups.

To the best of our knowledge, this is the first contemporary population-based study to evaluate the discriminative ability of advanced DL models for the prediction of axillary lymph node status in patients with cN0 breast cancer using only clinicopathological variables. Five ML models with increasing complexity and novelty, ranging from LR to Transformer, were compared. Advanced DL models, including ResNet and Transformer, demonstrated no significant improvement in overall performance compared with LR, and CatBoost and MLP also showed no improvement. Moreover, the improvement observed across all ML models compared to the univariable T-size model was minimal. These unconventional observations suggest that there is no clear nonlinear interaction among the 13 included clinical features, rendering them unsuitable for exploitation using nonlinear models. Consequently, the advantages of the DL architectures and their strategies are limited. However, with a predefined FNR of 10%, the DL models outperformed LR. This threshold was defined to address the estimated FNR of SLNB, which is the gold standard for evaluating the axillary nodal status.

The presented models demonstrated ROC AUCs of 0.704–0.712 and PR AUCs of 0.253–0.273 in the test set (Table 3), which were generally in accordance with the results of previous studies using only clinicopathological data but based on traditional ML techniques^22–24,26,57^. LR, MLP and CatBoost were used as benchmarks to offer a comparison between the proposed DL models and the traditional ML methods, since a direct comparison with existing research is challenging due to differences in study populations and predictive variables. First, lymphovascular invasion (LVI), which is difficult to evaluate accurately in a preoperative setting, was included in the prediction models^58^. Although LVI has been established as a critical factor in assessing the risk of nodal involvement, its microfocal nature presents challenges for accurate interpretation in the preoperative setting. This study focuses exclusively on clinicopathological variables that could be obtained preoperatively, either through imaging modalities for tumor size or core needle biopsy for tumor grade, biomarkers, and surrogate molecular subtypes. LVI was therefore excluded. Second, the study cohorts were older (1996–2012). Third, patients with more advanced tumors (T3) were included, and higher incidences of nodal metastasis (28–38%) related to larger primary tumors were observed. Importantly, unlike these studies, our prediction models were specifically designed to handle only clinicopathological variables that are readily accessible in a preoperative setting; therefore, LVI was not included, although it has previously been recognized as one of the strongest predictors of axillary nodal metastasis^45^. This approach enhances the practicality and versatility of our models, enabling their deployment in various settings and scenarios. Moreover, unlike most previously presented models for the prediction of nodal metastasis, which reported on the collection of micro- and macrometastases in sentinel and non-sentinel lymph nodes, our models were specifically trained to predict the presence of macro-SLNM. This is important since pure micro-SLNM (≤ 2.0 mm) is considered to be of minor clinical significance^59^, whereas for many patients with macro-SLNM, completion ALND and/or locoregional radiotherapy is still recommended^8,9^.

For all multivariable models, tumor size was the single most important predictor and showed a significant lead over the remaining predictors according to a post-hoc analysis of feature importance. Histological data and molecular profiles, including estrogen receptor (ER), PgR, HER2, and Ki67 expression, did not substantially enhance the accuracy of macro-SLNM prediction. This result explains the observation that the univariable T-size model exhibited competitive performance with the multivariable models trained on all features. This outcome is in accordance with previous findings recognizing tumor size as one of the most important clinicopathological predictors of axillary nodal status^45^.

To improve the model development, the five ML models were trained with 100 different hyperparameter settings (except for LR, which had 20 settings) and evaluated by fivefold cross-validation. Consequently, 2,100 models were evaluated (Supplementary Fig. S6). Despite the vast explored function space, the internal validation demonstrated low variance between the different models, suggesting that the uncertainty associated with the models and parameters was minimal^60^. Moreover, other popular DL strategies were employed to achieve efficient feature embedding and address imbalanced classification (Supplementary Figs. S4, S5). None of these attempts significantly boosted the performance except for the tokenizer for Transformer. This suggests that the primary source of prediction uncertainty stems from the data itself rather than the model or parameters. This argument is further supported by the individual interpretation based on the SHAP values, where patients with similar features exhibited close predictions, although they had divergent outcomes on SLNB. This implies the existence of unobserved key variables, rendering the target variable to appear random and unpredictable and points to data uncertainty encoded by the clinicopathological features. It is important to note that data uncertainty is inherently irreducible unless the input dimensionality is increased by incorporating additional variables with novel information into existing clinicopathological features.^61^

Accurate noninvasive prediction of macrometastatic lymph node status is important for improving the axillary management of patients with breast cancer. Detection at an earlier stage has decreased the node-positivity rate in newly diagnosed breast cancer^62^, and most patients do not benefit therapeutically from surgical axillary nodal staging. Because of its lack of effect on locoregional recurrence and breast-cancer-specific mortality^63,64^, the Choosing Wisely initiative^65^ has already declared that SLNB should no longer be routinely required for women ≥ 70 years of age with early-stage hormone receptor positive, HER2-negative cN0 invasive breast cancer. Accordingly, the American Society of Clinical Oncology guidelines on axillary management in breast cancer have been revised, endorsing the omission of SLNB for these patients after a case-by-case evaluation and patient-centered decision making^66^. Furthermore, the first results from the SOUND (Sentinel Node vs Observation After Axillary Ultra-Sound) trial recently showed that omission of axillary surgery is non-inferior with regard to 5-year distant disease-free survival in patients with T1 tumors and negative AUS results who are treated with breast-conserving surgery and radiotherapy, suggesting that selected patients can be safely spared routine SLNB^54^. In addition, ongoing randomized trials, e.g., INSEMA (Intergroup Sentinel Mamma)^67^ and BOOG 2013–08 (Dutch Breast Cancer Research Group)^68^ are currently evaluating the oncological safety of omitting routine SLNB in patients with cN0 early stage breast cancer and disease-free axillae on AUS. Although these trials are confined to breast-conserving surgery, they encompass patients across different age groups and molecular subtypes. Importantly, despite the widespread availability of genomic testing, chemotherapy remains a critical consideration, particularly for premenopausal women with ER + disease and nodal involvement, alongside endocrine therapy. Moreover, in patients with HER2-positive and triple-negative breast cancer undergoing upfront surgery, accurate nodal assessment is essential for tailoring adjuvant treatment regimens appropriately. Consequently, accurate noninvasive tools are needed to identify patients with cN0 breast cancer who are unlikely to benefit from surgical axillary nodal staging owing to the low risk of clinically relevant macro-SLNM.

Moreover, for patients with breast cancer undergoing mastectomy, preoperative noninvasive evaluation of the axillary nodal status is of particular interest. To improve postoperative quality of life^69^, patients undergoing mastectomy should be counselled about breast reconstruction options, and IBR should be offered to the vast majority, according to current guidelines^8,9^. Given that tumor size has a high predictive value for macro-SLNM, it is important to recognize that tumors tend to be larger in mastectomy cases compared to breast-conserving surgery. This may inherently increase the risk of nodal metastasis, leading to a greater likelihood of ALND or the need for radiotherapy in these patients. For patients with macro-SLNM, PMRT after IBR is associated with an increased risk of postoperative complications and reconstruction failure^10^. Assessing the need for PMRT is crucial for facilitating informed decision-making between patients and surgeons, particularly when considering breast reconstruction options, including the type and timing of the procedure.

Though this study indicates that a lack of nonlinear interaction among the clinicopathological variables limits the power of DL for detecting macro-SLNM, leveraging flexible feature engineering and advancements in computer vision and natural language processing (NLP), DL can demonstrate superior performance in clinical applications where heterogeneous tabular data and other modalities are available^70^. Recent research on diagnosis data comprising 172 features showed that Transformer and ResNet provided a definitive advantage over baseline models for various prediction tasks including hypertensive diseases, ischematic heart disease, diabetes, alcohol dependence and others^71^. In future studies, the integration of readily accessible preoperative imaging data with advanced DL techniques can be used to further enhance the performance of the prediction models. Studies combining clinicopathological characteristics with conventional ultrasonography^72^ or mammography^73^, utilizing radiomics—particularly deep radiomics^18,72^—have shown promising results in predicting axillary nodal status, despite the limited size of the study cohorts. Furthermore, hematoxylin and eosin-stained tissue sections of the primary breast tumor could be used, along with clinical data, to predict the risk of clinically important nodal metastasis. The first attempt to utilize this type of data with DL models was made based on the INSEMA cohort^74^. Although none of the presented image analysis algorithms showed better than random performance, the INSEMA cohort almost exclusively included low- to moderate-risk patients with hormone receptor-positive, HER2-negative luminal breast cancer, making it difficult to identify distinguishing image features. Finally, genomic data offer numerous opportunities to investigate gene signatures that could be included in prediction models to further improve the prediction of SLN metastasis^75^.

### Limitations of the study

This study has several limitations in addition to its retrospective nature. The study was conducted using registered data with a risk of misclassification. However, the NKBC database is a nationwide register recognized to have high coverage, with a < 5% proportion of missing values for most variables^76^. When cross-linked to the Swedish Cancer Register, the comparability was high, and excellent agreement with re-extraction of medical data was shown^76^. In our study, the average missing rate of all included predictors was approximately 2.3% (see Methods). Although missing data generally present challenges in the verification of a model, this is compensated for by the large sample size, which minimizes the effect of such errors, and meticulous data curation. Moreover, preprocessing steps, including missing value imputation, categorical embedding, and normalization, were conducted after separating the development and test sets. Therefore, information leakage from the test set is prevented. The possible variation in the prevalence of macro-SLNM between different patient populations may reduce the generalizability of the results for populations with markedly different proportions of node-positive breast cancer. However, procedures for adjusting the model predictions to a shifted a priori probability have been proposed^77^. Although the models were developed to predict the risk for macro-SLNM preoperatively and only variables that were feasible to obtain in a preoperative setting were included, the histopathological variables in the present study were collected from the final pathological evaluation. Even though histological grade, histopathological type and molecular profile can be readily assessed with high accuracy in the preoperative setting from routine core needle biopsy^58^, the tumor size and the presence of multifocality require thorough measurements across multiple imaging modalities to be accurately estimated^78–80^. Consequently, caution is warranted regarding potential differences between pre- and postoperative values. Therefore, further studies applying only preoperative variables should be conducted to validate these results.

In conclusion, this study extensively explored the capacity of DL models to predict macro-SLNM in patients with cN0 breast cancer using only clinicopathological characteristics. Under the constraint that the FNR is no more than 10%, which reflects the generally accepted FNR of SLNB^46^, Transformer was superior to the other models. This suggests that DL models hold promise for providing better noninvasive prediction of clinically important macrometastatic nodal status. Furthermore, the results in terms of AUCs, as well as feature analyses, suggest that inclusion of additional predictors would be essential for further improvement.

## Methods

### Ethical declaration and regulations

The research study and data usage agreements were reviewed and approved by the Swedish Ethical Review Authority (2019-02,139). The study was conducted using only data from the Swedish National Quality Register for Breast Cancer (NKBC). The need for informed consent was waived by the Swedish Ethical Review Authority for this register-based study in accordance with the national legislation. Construction and reporting of the prediction models followed the guidelines of Transparent Reporting of a Multivariable Prediction Model for Individual Prognosis or Diagnosis (TRIPOD)^81^.

### Study cohort

Data from all women diagnosed with breast cancer in Sweden between 2014 and 2017 who underwent surgery as primary treatment were retrospectively collected from the NKBC database. The exclusion criteria were as follows: bilateral breast cancer, neoadjuvant chemotherapy, ductal carcinoma in situ, tumor size > 50 mm or unknown, stage IV breast cancer, palpable axillary lymphadenopathy, incongruent or missing axillary nodal status data, and omission of SLNB. Patients diagnosed between 2014 and 2016 were allocated to the model development set (training and validation), whereas those diagnosed in 2017 were assigned to the temporal test set (test). To establish the internal training and validation sets, a fivefold cross-validation approach was applied to the development set.

### Outcome

The outcome of interest was the prediction of macro-SLNM, which was defined as the presence of ≥ 1 macrometastases in the SLNs. The identification of sentinel lymph nodes typically involves the use of tracers such as a radioactive isotope combined with blue dye or superparamagnetic iron oxide (SPIO). According to the American Joint Committee on Cancer classification criteria, nodal metastases were classified as macrometastatic if > 2 mm in diameter.^82^ Consequently, the ground-truth values of macro-SLNM were used to supervise the learning of prediction models.

### Predictor variables

Predictive variables were selected according to previous literature^42,43^ and previous results from our research group^14,24^. Information on lymph node status, patient characteristics, and tumor characteristics were retrieved from the NKBC. The features of interest were age, menstrual status, mode of detection (mammography screening or symptomatic presentation), number of invasive foci, invasive tumor stage, tumor size, Nottingham histological grade, histopathological type, and molecular profile (ER, PgR, HER2, Ki67, and St. Gallen surrogate molecular subtype). All histopathological variables (number of invasive foci, tumor size, histological grade, histopathological type and molecular profile) were assessed during the final pathological examination of the primary breast tumor and evaluated according to the Swedish Society of Pathology criteria^83^.

Histological types were categorized into three groups: NST, ILC, and other types of invasive carcinoma. The expressions of ER, PgR, and Ki67 were assessed by immunohistochemistry (IHC). Low, intermediate, and high expression of Ki67 was determined according to local cutoff values based on the lab-specific thresholds that were in use in Sweden during that time. To evaluate HER2 status, IHC and in situ hybridization (ISH) were performed, and tumors were classified as HER2-positive if they had IHC 3 + scoring and/or a positive ISH test. The classification of surrogate molecular subtypes—Luminal A-like, Luminal B-like, HER2-positive, and triple-negative breast cancer—was based on a modification of the St. Gallen 2019 guidelines and the classification proposed by Maisonneuve et al. (utilizing markers including ER, PR, HER2, Ki-67, and NHG), as previously reported (Supplementary Table S2).^84^ Invasive tumor stage was classified into T1 ($\le$ 20 mm), T2 (> 20 mm but ≤ 50 mm) and T3 ($>$ 50 mm), with only T1-T2 included in this study. A total of 26 variables derived from the 13 clinical features were used in the prediction models (Supplementary Table S1). ML models, especially DL models, have a good capacity for handling input redundancy, which allowed us to utilize redundant variables directly during model development and testing. However, when reporting statistical and feature importance analyses, we chose not to present redundant variables that could be derived from other variables.

### Univariable model

To investigate the predictive ability of the available variables, we first benchmarked a univariable model based solely on tumor size, as it was previously determined to be one of the most important predictors of nodal status.^45^ Tumor size was normalized to zero mean and unit variance. Subsequently, a sigmoid function was applied to generate scores ranging from 0 to 1, such that tumors with larger diameters were predicted to have a higher risk of macro-SLNM.

### Multivariable models

To enhance predictive ability, all preoperative variables were employed when training the five ML models: LR, MLP, ResNet, Transformer, and CatBoost. LR, linearly combining predictive variables, is a straightforward yet effective and robust approach. MLP comprises multiple layers of non-linear activation nodes, enabling it to handle non-linearly separable data. CatBoost was included as it is rapidly gaining popularity among gradient-boosted decision tree models because of its intrinsic support for categorical features and the ordered boosting technique, which helps overcome overfitting. These distinct characteristics have made LR, MLP and decision tree models widely employed in the medical domain, including for predicting axillary status^22–26^. On the other hand, ResNet, a fundamental component of contemporary DL networks, has achieved success in computer vision and NLP. The Transformer model, which relies on attention mechanisms, represents the cutting-edge architecture for large language models.

The model development and validation were conducted using Python (v3.8.8). The package dependencies were Scikit-learn (0.24.1) for LR and XGBoost (1.3.3) for CatBoost, whereas the DL models (MLP, ResNet, and Transformer) were built using PyTorch (1.13.1).

### Preprocessing

Categorical variables containing ordinal information were encoded as numbers to preserve their original relationships. Non-ordinal categorical variables were one-hot labeled. CatBoost employs ordered target statistic encoding for built-in categorical support^38^. Continuous features were normalized using the quantile transformation provided by the scikit-learn library. This approach effectively reduced the impact of outliers. In addition, tokenization, a common technique in NLP for learning meaningful word embeddings, was adapted for Transformer models applied to tabular data^35^. Therefore, to investigate its impact on performance, an embedding tokenizer module was implemented on top of the DL models. Missing numerical variables were imputed using the mean value, whereas missing categorical variables were imputed using the mode. In the overall study cohort, 14.5% of the patients had at least one missing value, and the average missing rate of all predictors was approximately 2.3%. All preprocessing steps, including missing-value imputation, categorical embedding, and normalization, were conducted separately for the development and test sets after splitting the data. Thus, information leakage from the test set was prevented.

### Imbalanced classification

To address the challenge of imbalanced distribution in macro-SLNM, advanced loss functions were implemented using the following strategies:

1. Weighted binary cross-entropy was achieved by introducing compensation weights to the macro-SLNM samples to alleviate the effect of being in the minority class.
2. Focal loss was employed to dynamically emphasize misclassified samples, thereby emphasizing more challenging cases.
3. Triplet loss was used to create balanced training samples by constructing triplet sets (anchor, positive, and negative samples based on their similarity or dissimilarity), thereby enhancing the ability of the model to discriminate between different classes.

### Hyperparameter optimization

To evaluate the searched models, the PR and ROC AUCs were calculated on the internal validation sets, and the best hyperparameters were selected based on a composite score combining the two metrics. The budget for tuning was set to 100 trials for all algorithms except LR, which used 20 trials to optimize a single parameter. The search space encompassed model parameters (such as embedding size, depth and width of neural networks, and dropout rates) and training parameters (such as learning rates and weight decay). Detailed descriptions of the default and hyperparameter search space can be found in Supplementary Section B. Hyperparameters were tuned using Bayesian optimization (the Tree-Structured Parzen Estimator algorithm) through the Optuna library (2.6.0),^85^ which has been shown to outperform random search.

### Evaluation

After determining the best hyperparameters or utilizing the default hyperparameters, the five multivariable models were trained on fivefold cross-validation splits. The performance of the models was evaluated using the test set by calculating the mean and standard deviation of the ROC and PR AUCs across the fivefold models. PR AUCs were calculated to reflect the imbalanced classification problem. Furthermore, the specificity, positive predictive value (or precision), NPV, and accuracy were reported at thresholds optimized for a sensitivity of 90%. This approach was adopted to minimize false-negative predictions.

### Feature importance

The feature importance of the developed models was estimated using the SHAP explainer^41^. For the logistic models, absolute model coefficients were used to approximate relative feature importance. The consistency between the two approaches was examined to ensure reliable and interpretable results. Based on the model that performed best under the constraint of sensitivity ≥ 90%, further SHAP explanations were applied at an individual level by random selection of true-positive, false-positive, true-negative, and false-negative predictions from the test set.

### Statistical analysis

Significant differences in patient and tumor characteristics were reported between the development and test sets, as well as between patients with and without macro-SLNM in the overall study cohort. Student’s *t* test was utilized to analyze differences in continuous variables, and the *χ*^2^ test was used for categorical variables. All statistical tests were two-tailed, and the significance level was set at *P* = 0.05. It is important to note that a statistically significant difference only indicates a difference at a certain level of confidence. It does not provide information on the magnitude or degree of the effect size. Therefore, we conducted an effect size analysis to address this issue. For continuous variables, the effect size was evaluated by the difference in means relative to the standard deviation, referred to as Cohen’s *d*^86^. A non-trivial effect size for continuous variables was defined as |*d*|≥ 0.50^87^. The effect size for categorical variables was evaluated using Cramer’s *V*^88^. Although the odds ratio is frequently employed to estimate effect size, it is limited to 2 × 2 confusion matrices. In our case, we have larger matrices, making Cramer’s *V* a more appropriate measure. A non-trivial effect size was defined as |*V*|≥ 0.30, ≥ 0.21, and ≥ 0.17 for 1, 2, and 3 degrees of freedom, respectively^44^. Data were analyzed between April 2023 and May 2023. All statistical analyses were performed using Python (v3.8.8).

## Supplementary Information

Supplementary Information.

## Data Availability

The data used in this study cannot be deposited in a public repository because of ethical prohibitions but are available from the lead contact upon reasonable request. An overview of NKBC data can be found at https://statistik.incanet.se/brostcancer/. All codes used for modeling are available at https://github.com/yandex-research/tabular-dl-revisiting-models.
