# Supplementary material for: Identification of sentinel lymph node macrometastasis in breast cancer by deep learning based on clinicopathological characteristics
Source: Sci Rep. 2024 Nov 6;14:26970. doi: 10.1038/s41598-024-78040-y (PMC11541545; doi:10.1038/s41598-024-78040-y)
Supplement: Supplementary file 1 — Supplementary Information. [file 41598_2024_78040_MOESM1_ESM.pdf]

Identification of Sentinel Lymph Node Macrometastasis in Breast Cancer by Deep Learning Based on Clinicopathological Characteristics

Author list:

Daqu Zhang[1], Miriam Svensson[2], Patrik Edén[1] Looket Dihge[2,3*]

Affiliations:

1. Center for Environmental and Climate Science, Division of Computational Science for Health and Environment, Lund University, Lund, Sweden

2. Department of Clinical Sciences Lund, Division of Surgery, Lund University, Lund, Sweden.

3. Department of Plastic and Reconstructive Surgery, Skåne University Hospital, Malmö, Sweden.

[*]**Correspondence**:

Department of Clinical Sciences, Division of Surgery, Lund University, 223 81 Lund, Sweden

E-mail: looket.dihge@med.lu.se

Telephone: +46 70 255 68 35

Supplementary:

Table S1. Mapping of all variables included in the prediction models

Clinical features	Predictors	Value space
Age	Age (y)	(0, +∞)
Menstrual status	Menstrual status<br>Premenopausal at diagnosis	[Premenopausal, Postmenopausal]<br>[No, Yes]
Mode of detection	Mode of detection	[Symptomatic presentation, Mammographic screening]
Number of invasive foci	Number of invasive foci<br>Multifocality	(0, +∞)<br>[No, Yes]
T-stage	T-stage	[T1, T2]
Tumor size	Tumor size (mm)	(0, 50)
Histological type (classification 1)	Histological type classification 1<br>Histological type classification 2<br>Histological type classification 3	[NST, ILS, Others]<br>[NST, ILS, Other or mixed]<br>[NST or ILS, Others]
Histological grade	Histological grade<br>Histological grade = III	[1, 2, 3]<br>[No, Yes]
ER%	ER%<br>ER >1%<br>ER >10%	(0, 100)<br>[No, Yes]<br>[No, Yes]
PgR%	PgR%<br>PgR >1%<br>PgR >10%<br>PgR >20%	(0, 100)<br>[No, Yes]<br>[No, Yes]<br>[No, Yes]
Ki67%	Ki67%<br>Ki67 status<br>Ki67 >20%	(0, 100)<br>[Low, Intermediate, High]<br>[No, Yes]
HER2 status	HER2 status	[Negative, Positive]
St Gallen surrogate molecular subtype	St Gallen surrogate molecular subtype<br>TNBC	[LumA, LumB, HER2+, TNBC]<br>[No, Yes]

A total of 26 predictors derived from 13 clinical features were used in the prediction models. When reporting statistical and feature importance analyses, we only reported the 13 clinical features presented in the left column.

NST, no special type; ILC, invasive lobular carcinoma; ER, estrogen receptor; PgR, progesterone receptor; HER2, human epidermal growth factor receptor 2; LumA, luminal A-like; LumB, luminal B-like; HER2+, HER2-positive; TNBC, triple-negative breast cancer.

Table S2. The definition of surrogate molecular subtypes

Definition of surrogate molecular subtypes[a]	
Luminal A-like<br>(ER+/HER2-)[b]	NHG 1<br>NHG 2 and Ki67 low<br>NHG 2 and Ki67 intermediate and PR ≥20%
Luminal B-like<br>(ER+/HER2-)[b]	NHG 3<br>NHG 2 and Ki67 high<br>NHG 2 and Ki67 intermediate and PR <20%
HER2 positive[b]	HER2+
Triple-negative[b]	ER- and PR- and HER2-

[a]The definition of molecular surrogate subtypes was based on a modification of the St. Gallen 2019 guidelines and the classification proposed by Maisonneuve et al [S1].

[b]Handling of missing data:

HER2 positive: Only HER2 status required for classification. Hence, cases with missing data for one or more of the variables ER, PR, Ki67 and NHG were classified if HER2 status was available.

Triple-negative: Complete data for ER, PR and HER2 required. Hence, cases with missing data for Ki67 and/or NHG were classified.

Luminal: NHG (Nottingham Histological Grade) required for classification. Cases with missing Ki67 classified only if NHG 1 or NHG 3. Cases with missing PR classified only if NHG 1, NHG 3 or NHG2 with low or high Ki67.

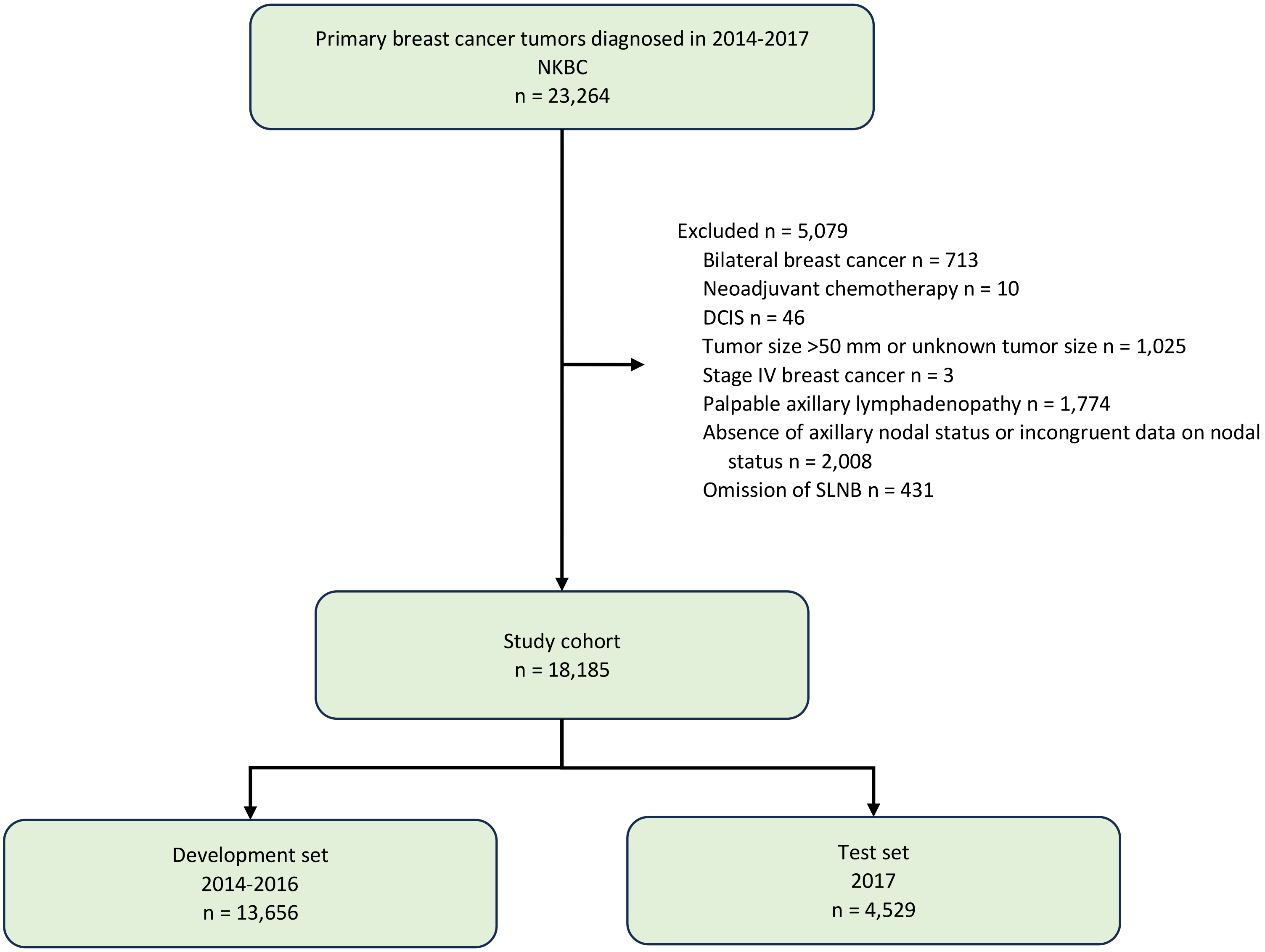

Figure S1. **Patient selection process**

NKBC, Swedish National Quality Register for Breast Cancer; DCIS, ductal carcinoma *in situ*; SLNB, sentinel lymph node biopsy; cN0, clinically node-negative.

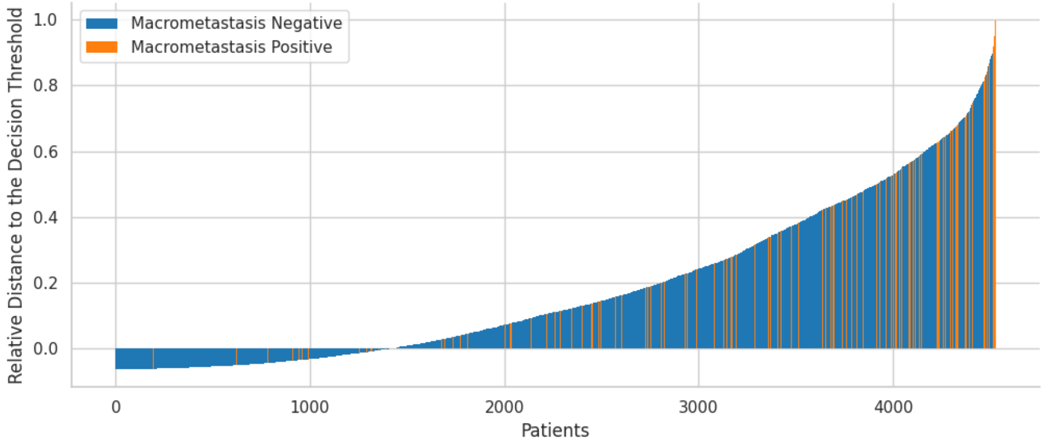

Figure S2. Relative distance to the decision threshold on individual predictions by Transformer in the test set

The threshold for positive *vs* negative macrometastatic sentinel lymph node status was optimized at a sensitivity of 90%, addressing the generally accepted false-negative rate of sentinel lymph node biopsy of 10% [S2]. Under this constraint, the threshold was 0.062. From left to right, patients from the test set are sorted by increasing predicted probability of sentinel lymph node macrometastasis as predicted by Transformer.

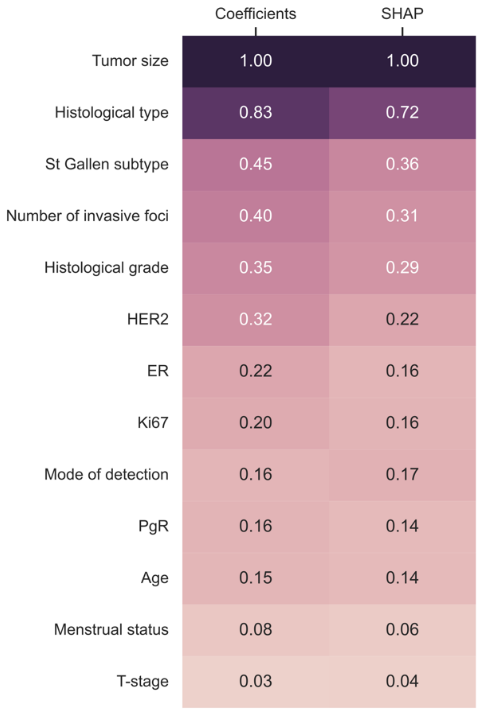

Figure S3. Comparison between feature importance estimation of logistic regression (LR) coefficients and Shapley Additive exPlanation (SHAP) values

The predictors are ranked by their average importance, with decreasing values from top to bottom. The Pearson correlation coefficient between the LR coefficients and LR SHAP values was 0.99.

HER2, human epidermal growth factor receptor 2; ER, estrogen receptor; PgR, progesterone receptor.

Section A: Deep learning (DL) strategies

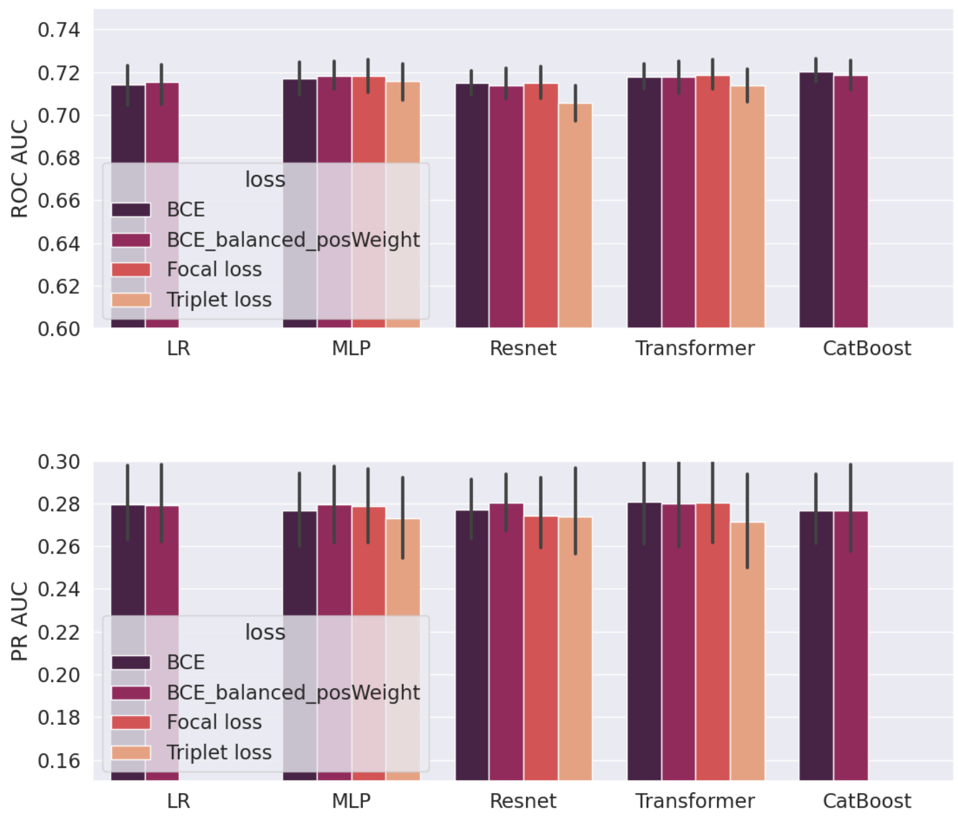

Figure S4. Predictive performance of models trained with advanced losses evaluated by 5-fold cross validation.

For the loss of binary cross-entropy (BCE) with balanced weights in the positive class, *pos_weight* was set to 5, which was approximately the ratio of negative to positive samples. The focal loss had two parameters that were set to the recommended values (*gamma* = 2, *alpha* = 0.25) according to the original paper[S3]. Triplet loss parameters were set to their default values (*margin* = 1.0, p = 2). Compared with vanilla BCE, BCE with balanced weights (Paired *t* test: receiver operating characteristic [ROC], *P* = 0.90; precision recall [PR], *P* = 0.25) and focal loss (Paired *t* test: ROC, *P* = 0.21; PR, *P* = 0.63) showed no significant improvement, and triplet loss

(Paired *t* test: ROC, 0.71 *vs* 0.72, *P* = 0.005; PR, 0.27 vs 0.28, *P* = 0.014) showed inferior performance. Data are represented as mean ± standard deviation in the plot.

AUC, area under the curve; LR, logistic regression; MLP, multilayer perceptron.

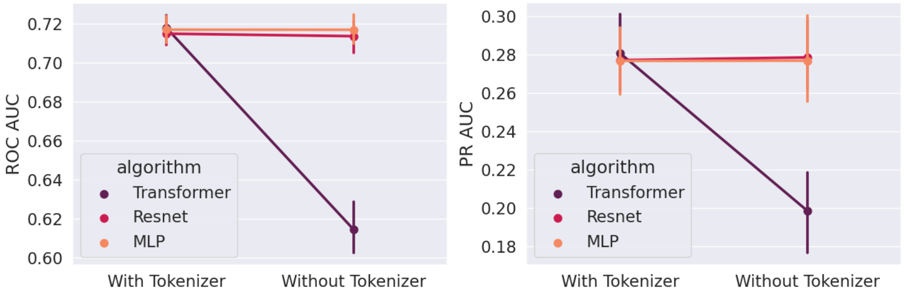

Figure S5. Performance of deep learning models with and without feature tokenizers evaluated by 5-fold cross validation.

A feature tokenizer is a successful approach for feature embedding in nature language processing. The tokenizer was an indispensable component of Transformer, but it had little effect on multilayer perceptron (MLP) and ResNet. Data are represented as mean ± standard deviation.

ROC, receiver operating characteristics; AUC, area under the curve.

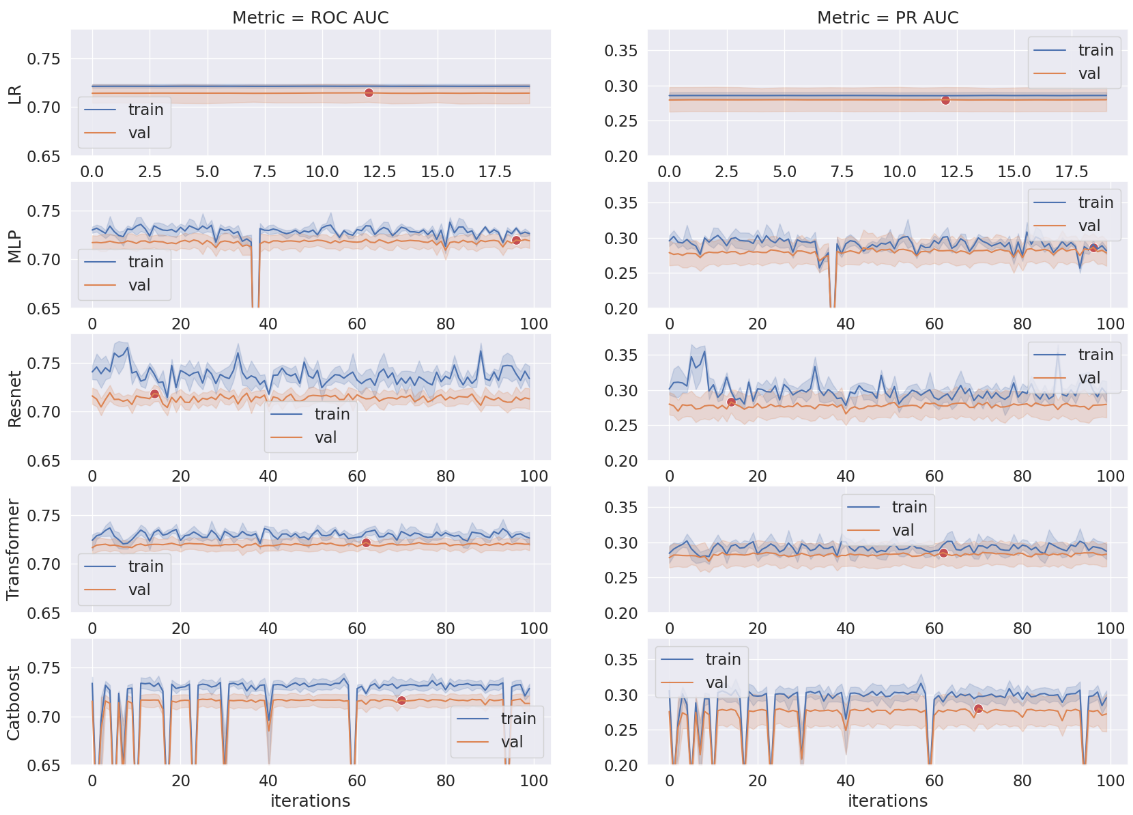

Figure S6. Performance of the developed algorithms evaluated by 5-fold cross validation during the hyperparameter search process

Overall, no hyperparameter was found to significantly outperform the others in the development set across the five algorithms. Logistic regression (LR) performed almost the same on the development set for various regularization strengths. For multilayer perceptron (MLP), the validation performance remained largely consistent across most of the hyperparameter space, with only one iteration showing complete failure. ResNet displayed a tendency toward overfitting compared to the other algorithms, but no exceptional parameters were identified. Transformer demonstrated robustness against hyperparameter tuning and overfitting, although no superior hyperparameters were identified. However, CatBoost proved to be sensitive to hyperparameter

tuning and experienced frequent failures, indicating that no optimal parameters have yet been found.

ROC, receiver operating characteristic; AUC, area under the curve; PR, precision recall.

Section B: Hyperparameter Search Space

Logistic regression (LR)

We fixed and did not optimize the following hyperparameters:

- *max-iter* = 2000
- *n-jobs* = 10

The only tuned hyperparameter was C, which is the inverse of the regularization strength. The search distribution was Uniform [0.5,1.5], and the default value was 1.0.

CatBoost

We fixed and did not optimize the following hyperparameters:

- early-stopping-rounds = 50
- iterations = 2000
- metric-period = 10
- od-pval = 0.001

Search space used for Optuna and default values for LR

Parameter	Search Space	Default Value
Bagging temperature	Uniform [0, 1]	1
Max depth	UniformInt [3, 10]	6
L2 leaf reg	LogUniform [1, 10]	3
Leaf estimation iterations	UniformInt [1, 10]	None
Learning rate	LogUniform [1e-5, 1]	0.04231

Multilayer perceptron (MLP)

We fixed and did not optimize the following hyperparameters:

- optimizer = 'adamw'
- patience = 16
- batch-size = 256

Search space used for Optuna and default values for MLP

Parameter	Search Space	Default Value
Categorical embedding size	UniformInt [32, 128]	64
# Layers	UniformInt [1, 4]	2
Layer size	UniformInt [1, 256]	[32, 128]
Dropout	{0, Uniform [0, 0.5]}	0.1
Weight decay	{0, LogUniform [1e-6, 1e-3]}	1e-5
Learning rate	LogUniform [1e-5, 1e-2]	1e-4

ResNet

We fixed and did not optimize the following hyperparameters:

- optimizer = 'adamw'
- patience = 16
- batch-size = 256

Search space used for Optuna and default values for ResNet

Parameter	Search Space	Default Value
Categorical embedding size	UniformInt [32, 128]	64
# Layers	UniformInt [1, 4]	3
Layer size	UniformInt [16, 256]	96
Hidden factor	Uniform [1, 4]	2
Hidden dropout	Uniform [0, 0.05]	0.2
Residual dropout	{0, Uniform [0, 0.5]}	0.2
Weight decay	{0, LogUniform [1e-6, 1e-3]}	1e-5
Learning rate	LogUniform [1e-5, 1e-2]	1e-3

Transformer

- optimizer = 'adamw'
- patience = 16

- batch-size = 256
- n-heads = 4
- initialization = ‘kaiming’
- prenormalization = true

Search space used for Optuna and default values for Transformer

Parameter	Search Space	Default Value
Categorical embedding size	UniformInt [32, 128]	64
# Layers	UniformInt [1, 4]	1
FFN factor	Uniform [1, 4]	1.3
FFN dropout	Uniform [0, 0.05]	0.1
Attention dropout	Uniform [0, 0.05]	0.2
Residual dropout	{0, Uniform [0, 0.2]}	0
Weight decay	{0, LogUniform [1e-6, 1e-3]}	1e-5
Learning rate	LogUniform [1e-5, 1e-3]	1e-4

FFN, feedforward network.

Supplemental reference:

1. Maisonneuve, P. et al. Proposed new clinicopathological surrogate definitions of luminal A and luminal B (HER2-negative) intrinsic breast cancer subtypes. Breast Cancer Res. 16, 1–9 (2014).
2. D Krag, D. N. et al. Technical outcomes of sentinel-lymph-node resection and conventional axillary-lymph-node dissection in patients with clinically node-negative breast cancer: results from the NSABP B-32 randomised phase III trial. Lancet Oncol. 8, 881–888 (2007).
3. Lin, T.-Y., Goyal, P., Girshick, R., He, K. & Dollár, P. Focal loss for dense object detection. in Proceedings of the IEEE international conference on computer vision 2980–2988 (2017).
